# Surgical Management of Focal Chondral Defects of the Talus: A Bayesian Network Meta-analysis

**DOI:** 10.1177/03635465211029642

**Published:** 2021-09-20

**Authors:** Filippo Migliorini, Nicola Maffulli, Hanno Schenker, Jörg Eschweiler, Arne Driessen, Matthias Knobe, Markus Tingart, Alice Baroncini

**Affiliations:** † Department of Orthopaedic, Trauma, and Reconstructive Surgery, RWTH University Hospital, Aachen, Germany; ‡ Department of Medicine, Surgery and Dentistry, University of Salerno, Baronissi (SA), Italy; § Queen Mary University of London, Barts and the London School of Medicine and Dentistry, Centre for Sports and Exercise Medicine, Mile End Hospital, London, England; ‖ School of Pharmacy and Bioengineering, Keele University Faculty of Medicine, Stoke on Trent, England; ¶ Department of Orthopedics and Trauma Surgery, Lucerne Cantonal Hospital, Lucerne, Switzerland; Investigation performed at RWTH University Clinic, Aachen, Germany

**Keywords:** talus, chondral defect, AMIC, OAT

## Abstract

**Background:**

No consensus has been reached regarding the optimal surgical treatment for focal chondral defects of the talus.

**Purpose:**

A Bayesian network meta-analysis was conducted to compare the clinical scores and complications of mosaicplasty, osteochondral auto- and allograft transplant, microfracture, matrix-assisted autologous chondrocyte transplant, and autologous matrix-induced chondrogenesis (AMIC) for chondral defects of the talus at midterm follow-up.

**Study Design:**

Bayesian network meta-analysis; Level of evidence, 4.

**Methods:**

This Bayesian network meta-analysis followed the PRISMA (Preferred Reporting Items for Systematic Reviews and Meta-Analyses) extension statement for reporting of systematic reviews incorporating network meta-analyses of health care interventions. PubMed, Embase, Google Scholar, and Scopus databases were accessed in February 2021. All clinical trials comparing 2 or more surgical interventions for the management of chondral defects of the talus were accessed. The outcomes of interest were visual analog scale (VAS) score, American Orthopaedic Foot and Ankle Society (AOFAS) score, rate of failure, and rate of revision surgery. The network meta-analysis were performed through the routine for Bayesian hierarchical random-effects model analysis. The log odds ratio (LOR) effect measure was used for dichotomous variables, and the standardized mean difference (SMD) was used for continuous variables.

**Results:**

Data from 13 articles (521 procedures) were retrieved. The median length of the follow-up was 47.8 months (range, 31.7-66.8 months). Analysis of variance revealed no difference between the treatment groups at baseline in terms of age, sex, body mass index, AOFAS score, VAS score, and mean number of defects. AMIC demonstrated the greatest AOFAS score (SMD, 11.27) and lowest VAS score (SMD, –2.26) as well as the lowest rates of failure (LOR, 0.94) and revision (LOR, 0.94). The test for overall inconsistency was not significant.

**Conclusion:**

At approximately 4 years of follow-up, the AMIC procedure for management of focal chondral defects of the talus produced the best outcome.

Focal chondral defects of the talus are common.^35^ Given the limited intrinsic regeneration capability of cartilage, surgical intervention can be necessary.^15,30^ If left untreated, patients can experience increasing pain, reduced quality of life, reduced sporting activity, and early osteoarthritis.^29^ Traditionally, microfracture has been considered the first-line intervention for focal chondral defects of the talus.^13^ Several osteochondral transplant procedures have been proposed. With osteochondral autograft or allograft transplant (OAT), an osteochondral graft is harvested from a nonweightbearing zone of the knee and transplanted to fill the chondral defect of the talus.^1,16^ With mosaicplasty, several osteochondral grafts or plugs are harvested from a nonweightbearing zone of the knee to produce a mosaic-like structure.^37^ In matrix-assisted autologous chondrocyte transplantion (MACT), chondrocytes harvested from a nonweightbearing area of the knee are cultivated, expanded, and implanted into a membrane.^27,28^ In a second surgical session, the chondrocyte-loaded membrane is transplanted into the defect with custom-made instruments in a full arthroscopic fashion.^34^ Recently, autologous matrix-induced chondrogenesis (AMIC) has been proposed for the management of chondral defects of the talus.^3,5^ AMIC exploits the regenerative potential of bone marrow–derived cells.^9,17^

Despite all these options, it is unclear which is the optimal surgical treatment for focal chondral defects of the talus, and no consensus has been reached. To the best of our knowledge, no network meta-analysis has been conducted previously to evaluate the treatments for talar chondral defects. Therefore, a Bayesian network meta-analysis was conducted to compare these strategies for the surgical management of chondral defects of the talus at midterm follow-up. The present study compared the efficacy of these strategies in terms of clinical scores and complications.

## Methods

### Search Strategy

This Bayesian network meta-analysis followed the PRISMA (Preferred Reporting Items for Systematic Reviews and Meta-Analyses) extension statement for reporting of systematic reviews incorporating network meta-analyses of health care interventions.^25^ Before the literature search was conducted, the PICOT framework was established as follows:

- P (Problem): focal chondral defect of the talus
- I (Intervention): surgical management
- C (Comparison): AMIC, OAT, microfracture, mosaicplasty, MACT
- O (Outcomes): clinical scores and complications
- T (Timing): ≥18 months of follow-up

### Data Source

Two authors (F.M., A.B.) conducted the literature search independently. PubMed, Embase, Google Scholar, and Scopus databases were accessed in February 2021. The following keywords were used in combination: *ankle, talus, cartilage, damage, chondral, articular, injury, chondropathy, focal, defect, pain, membrane, autologous matrix-induced chondrogenesis, matrix-assisted autologous chondrocyte transplantation, MACT, AMIC, OAT, cylinder, osteochondral, transplantation, outcomes, microfractures, failures, surgery, management, allograft, autograft, failure, revision*. The same authors independently screened the resulting articles. The full-text versions of the articles of interest were accessed, and a cross-reference of the bibliographies was conducted. Divergences were solved by a third author (N.M.).

### Eligibility Criteria

All clinical trials that compared 2 or more surgical interventions for the management of talar chondral defects were accessed. Given the authors’ language abilities, articles in English, German, Italian, French, and Spanish were eligible. Only studies of level I to IV of evidence, according to the Oxford Centre of Evidence-Based Medicine,^24^ were considered. Other eligibility criteria were studies that focused on AMIC, OAT, microfracture, MACT, and mosaicplasty; involved patients with a focal lesion of the talus; reported data on a minimum of 10 patients; clearly stated the type of intervention; had a minimum of 18 months of follow-up; and reported quantitative data under the outcomes of interest. Not eligible were studies involving patients with end-stage joint osteoarthritis or patients with kissing lesions; animal, computational, or biomechanical studies; and studies augmenting the procedures with less committed cells (eg, mesenchymal stem cells).

### Data Extraction

Two authors (F.M., A.B.) separately performed data extraction. Study details such as author, year, journal, study design, and length of follow-up were retrieved. Data regarding patient characteristics at baseline were collected: number of procedures, defect size, patients’ mean age and body mass index (BMI), and patients’ sex. Also retrieved were visual analog scale (VAS) and American Orthopaedic Foot and Ankle Society (AOFAS)^41^ scores and the rates of failure and revision surgeries. Failure was defined as recurrence of pain and/or catching symptoms, graft hypertrophy, and/or partially or completely displaced delamination seen on magnetic resonance imaging (MRI) or arthroscopic examination.^23,31,32^

### Methodological Quality Assessment

Two independent authors assessed methodological quality (F.M., A.B.) using the risk of bias graph tool of the Review Manager software (The Nordic Cochrane Collaboration). The following factors pertaining to risk of bias were evaluated: selection, detection, reporting, attrition, and other source of bias.

### Statistical Analysis

The statistical analysis was performed by the main author (F.M.), using STATA software/MP (Stata Corporation). To assess baseline data, the Shapiro-Wilk test was performed to investigate data distribution. For parametric data, mean and standard deviation were evaluated. Baseline comparability of parametric data was assessed using analysis of variance (ANOVA), with *P* > .1 considered satisfactory. For nonparametric data, median and interquartile range were evaluated. The baseline comparability of nonparametric data was assessed by the Kruskal-Wallis test, with *P* > .1 considered satisfactory. Network meta-analysis were performed through the STATA routine for Bayesian hierarchical random-effects model analysis. The inverse variance method was used for all of the comparisons. The log odds ratio (LOR) effect measure was used for dichotomous variables, whereas the standardized mean difference (SMD) was used for continuous variables. Overall inconsistency was evaluated through the equation for global linearity via the Wald test. If the *P* value was >.1, the null hypothesis could not be rejected, and the consistency assumption was accepted at the overall level of each treatment. All variables were compared in the network analyses against a fictitious group control: no event for binary comparisons and the maximal value of a score for continuous endpoints. For each outcome of interest, we constructed edge, interval, and funnel plots. Edge plots demonstrate the amount of a direct comparison and its weight in the overall network analysis. Interval plots rank the estimate effect related to each treatment resulting from the network comparisons. For the interval plots, both confidence intervals and percentile intervals were set at 95%. Funnel plots investigate the risk of bias of the outcome of interest considered; greater asymmetry in the plot was associated with a greater risk of bias.

## Results

### Search Result

**Figure 1.**
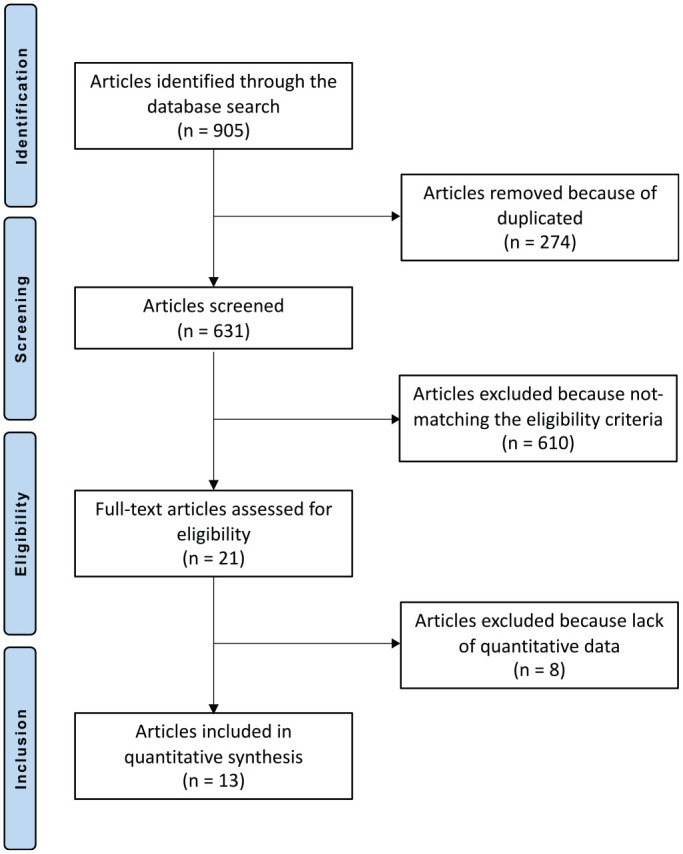
Flowchart of the literature search using the PRISMA (Preferred Reporting Items for Systematic Reviews and Meta-Analyses) guidelines.

The literature search resulted in 905 articles; of them, 274 were duplicates. A further 610 articles were not compatible with the eligibility criteria: noncomparative study (n = 309), not focused on talus (n = 192), short follow-up (n = 8), included kissing lesion (n = 7), not focused on focal defect (n = 12), combined with stem cells (n = 10), language limitations (n = 2), reported on other surgical interventions (n = 51), and other (n = 19). A further 8 studies did not report quantitative data under the outcomes of interest. This left 13 articles for inclusion in the present study. The literature search results are shown in Figure 1.

### Methodological Quality Assessment

**Figure 2.**
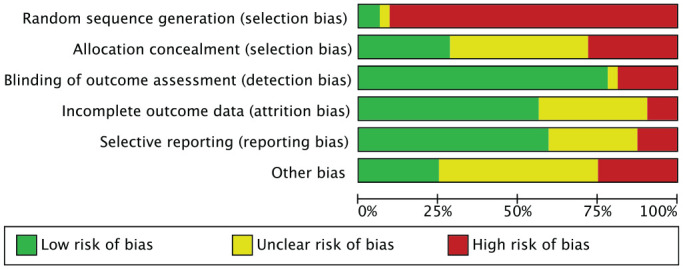
Methodological quality assessment.

Given the large number of retrospective comparative studies (10/13), the risk of selection bias was high. Assessor blinding was performed in 10 of the 13 studies, resulting in a low risk of detection bias. The risks of attrition bias and reporting bias were moderate to low, as were the risks of other bias. The overall score for risk of bias was low, attesting that this study had moderate to good methodological quality. The risk of bias graph is shown in Figure 2.

### Patient Characteristics

Data on 521 procedures were retrieved. The median length of follow-up was 47.8 months (range, 31.7-66.8 months). Of all patients, 42.4% (221/521) were women. The mean ± SD age of the patients was 35.0 ± 8.0 years, and the mean BMI was 25.1 ± 1.1. The mean defect size was 2.0 ± 1.6 cm^2^. At baseline, the mean VAS score was 7.4. ± 0.9 and the mean AOFAS score was 47.0 ± 8.2. The ANOVA revealed no difference between the treatment groups at baseline in terms of mean age and BMI, patient sex, defect size, and VAS and AOFAS scores (*P* > .1). Details of the studies are shown in Table 1.

**Table 1:** Details of the Included Studies^*a*^

Lead Author (Year)	Journal	Design	Treatment	Follow- up, mo	Procedures, n	Female Patients, %	Patients’ Mean Age, y
Ahmad^2^ (2016)	*Foot Ankle Int*	Randomized	OAT: allograft	40.5	16	37.5	39.7
			OAT: autograft	35.2	20	45.0	41.3
Apprich^4^ (2012)	*Osteoarthritis Cartilage*	Retrospective	MACT	48.0	10	60.0	31.0
			MFX	59.6	10	40.0	32.4
Becher^7^ (2019)	*Knee Surg Sports Traumatol Arthrosc*	Retrospective	MFX	67.2	16	56.3	33.3
			AMIC	68.4	16	56.3	32.4
D’Ambrosi^9^ (2017)	*Arthroscopy*	Retrospective	AMIC	27.0	17	52.9	25.0
			AMIC		14	26.0	47.0
Domayer^12^ (2012)	*Osteoarthritis Cartilage*	Retrospective	MFX	113.8	10	55.6	30.8
			MACT	65.4	10	77.8	25.4
Gobbi^18^ (2006)	*Arthroscopy*	Prospective	MFX	53.0	10	40.0	24.0
			Control group		11	45.5	32.0
			OAT: autograft		12	33.3	27.8
Gül^20^ (2016)	*J Foot Ankle Surg*	Retrospective	OAT: autograft	30.5	15	33.3	32.6
			OAT: autograft	28.9	13	8.3	36.7
Guney^21^ (2016)	*Knee Surg Sports Traumatol Arthrosc*	Prospective	MFX	47.3	19	37.4	47.4
			Control group	40.4	22	43.9	50.0
			Mosaicplasty	30.1	13	37.6	15.4
Haleem^22^ (2014)	*Am J Sports Med*	Retrospective	OAT: autograft	93.0	14	50.0	42.8
			OAT: autograft	85.3	28	39.3	44.1
Park^33^ (2018)	*Am J Sports Med*	Retrospective	OAT: autograft	71.4	18	41.6	
			OAT: autograft		28		
Shimozono^38^ (2018)	*Am J Sports Med*	Retrospective	OAT: autograft	52.0	63	42.9	36.0
			OAT: autograft	45.0	31	32.3	34.0
Shimozono^38^ (2018)	*Bone Joint Surg Am*	Retrospective	OAT: autograft	26.3	25	64.0	38.4
			OAT: allograft	22.3	16	37.5	43.6
Yoon^44^ (2014)	*Am J Sports Med*	Retrospective	OAT: autograft	45.0	22	31.8	37.1
			MFX	50.0	22	18.2	41.6

a AMIC, autologous matrix-induced chondrogenesis; MACT, matrix-assisted autologous chondrocyte transplant; MFX, microfracture; OAT, osteochondral autograft or allograft transplant.

### Outcomes of Interest

**Figure 3.**
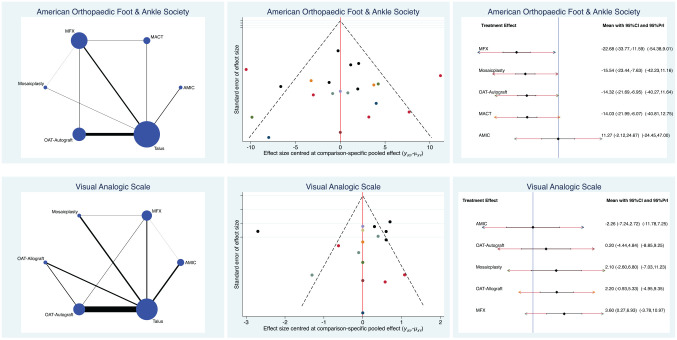
Edge, funnel, and interval plots of scores: American Orthopaedic Foot and Ankle Society and visual analog scale. AMIC, autologous matrix-induced chondrogenesis; MACT, matrix-assisted autologous chondrocyte transplantation; MFX, microfracture; OAT, osteochondral autograft or allograft transplant; PrI, percentile interval.

AMIC produced the highest AOFAS scores (SMD, 11.27; 95% CI, –2.12 to 24.67) and lowest VAS scores (SMD, –2.26; 95% CI, –7.24 to 2.72). The test for overall inconsistency was not significant (*P* = .6). Edge, funnel, and interval plots of the scores are shown in Figure 3.

### Complications

**Figure 4.**
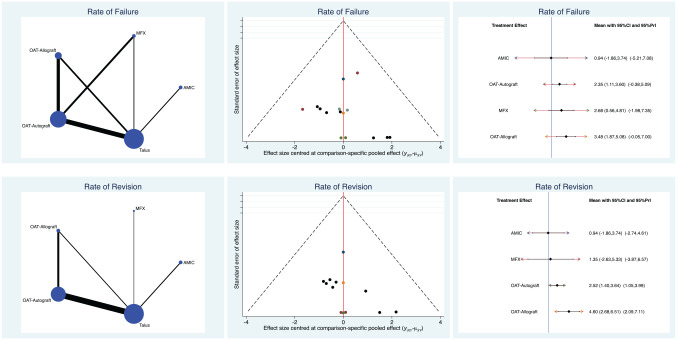
Edge, funnel, and interval plots of complications: failure and revision. AMIC, autologous matrix-induced chondrogenesis; MFX, microfracture; OAT, osteochondral autograft or allograft transplant; PrI, percentile interval.

AMIC demonstrated the lowest rates of failure (LOR, 0.94; 95% CI, –1.86 to 3.74) and revision (LOR, 0.94; 95% CI, –1.86 to 3.74). OAT evidenced the highest rates of failure (LOR, 3.48; 95% CI, 1.87 to 5.08) and revision (LOR, 4.60; 95% CI, 2.68 to 6.51). The test for overall inconsistency was not significant (*P* = .8). Edge, funnel, and interval plots of complications are shown in Figure 4.

## Discussion

The present Bayesian network meta-analysis demonstrated that AMIC for the management of focal osteochondral lesions of the talus performed better overall at approximately 4 years of follow-up. Patients undergoing OAT with allograft experienced the highest rate of complications, whereas microfracture resulted in the lowest values of patient-reported outcome measures.

To the best of our knowledge, this is the first Bayesian network meta-analysis to rank the treatments for talar chondral defects. AMIC exploits the potential of autologous bone marrow–derived mesenchymal stem cells, avoiding the harvesting of nonweightbearing cartilage, cell culture, and expansion. Moreover, being a single-stage procedure, it may present an attractive option for both patients and surgeons. The available scientific literature lacks head-to-head studies that compare AMIC with other surgical techniques for the management of knee chondral defects. Becher et al^7^ compared AMIC versus microfracture in a cohort of 32 patients (16 patients each group) with a minimum follow-up of 5 years, evidencing no differences in outcome. D’Ambrosi et al,^9^ using AMIC in a cohort of 17 young versus 14 old patients, concluded that AMIC was a reliable procedure regardless of patient age and that clinical outcome may depend on the preoperative conditions of the ankle. Walther et al^42^ performed a systematic review and meta-analysis including 12 studies (492 procedures). Although their analysis was affected by a high grade of heterogeneity, they evidenced a statistically significant improvement of AOFAS, VAS, and Foot Function Index scores.^8^ Given the lack of quantitative data, return to sports was not included in the network comparisons. D’Ambrosi et al^10^ retrospectively analyzed return to sports in a cohort of patients after AMIC: 80.8% (21/26) of patients returned to sports within 42.6 months.

Our network comparison indicated that autografts for osteochondral transplant performed better than allografts. These observations agree with previous head-to-head studies that compared the 2 grafts. Ahmad and Jones^2^ compared autograft versus allograft in a cohort of 40 patients in a randomized clinical trial. The results were comparable, but allografts had lower healing rates. Shimozono et al^38^ found better clinical and MRI outcomes and a lower rate of failure in the allograft group in a cohort of 25 patients. Allografts have been introduced to avoid harvesting morbidity, with initially promising results.^14^ However, allografts may deteriorate over time.^43^ MRI studies show that allografts are not properly incorporated in some patients, and cartilage fissures or cysts close to the host-graft interface are more frequent when allografts are used versus autografts.^14,19^ Further studies are required to clearly establish the pros and cons of both type of osteochondral transplant.

The present network meta-analysis is not without limitations. The most important limitations are the retrospective nature of the design and the overall poor quality of many of the included studies. The analyses were performed regardless of the surgical approach (arthroscopy, mini-arthrotomy, arthrotomy), the nature of the membrane (collagen or hyaluronic acid), the fixation methods (glue, fibrin, both, none), and the location of the lesion on the articular surface. Given the lack of comparative studies, autologous chondrocyte implant and particulated juvenile articular cartilage techniques were not included for analysis. Because of the limited available data, articles were considered regardless of the cause of the chondral defect, and further differentiation between primary and revision settings was not possible. The lack of quantitative data prevented comparison of the time to return to sports. Most authors reported data of interventions combined with other surgical procedures, such as osteotomy or ligament repair. Given the lack of data concerning complications, it was not possible to separately analyze the causes of failure. The heterogeneous procedures and the limited available data precluded evaluation of the use of mesenchymal stem cells.

Regenerative medicine is rapidly evolving through the use of mesenchymal stem cells and via better understanding of the cellular and molecular basis of hyaline cartilage healing. Future studies should overcome the current obstacles to clinical translation—namely, cell source, cell isolation, and expansion and differentiation methods. In the past few years, several protocols for cell processing have been developed, but no consensus has been reached. Deeper understanding of the interactions between mesenchymal stem cells and the microenvironment, related signaling patterns, and influence on the regenerative cascade is required to develop appropriate therapeutic protocols. Given these controversies, studies using mesenchymal stem cells were not included. The AOFAS score is one of the most commonly used scores to assess foot and ankle ailments.^36,40^ However, whether the AOFAS score is a valid and reliable measure for assessment is controversial.^6,11,26,39^ In view of these limitations, results must be interpreted with caution.

## Conclusion

At approximately 4 years of follow-up, AMIC displayed the most reliable results for the management of focal chondral defects of the talus.
